# The Potential of Cardiac Biomarkers in Differentiating Disease Subtypes in Patients with Systemic Sclerosis: Focus on GDF15, MR-pro ANP, and suPAR

**DOI:** 10.3390/ijms26093938

**Published:** 2025-04-22

**Authors:** Olga Gumkowska-Sroka, Anna Chudek, Aleksander Owczarek, Kornelia Kuźnik-Trocha, Kacper Kotyla, Jan Kurdybacha, Jerzy Chudek, Katarzyna Komosińska-Vassev, Katarzyna Winsz-Szczotka, Krystyna Olczyk, Przemysław Kotyla

**Affiliations:** 1 Department of Internal Medicine Rheumatology and Clinical Immunology, Faculty of Medical Sciences in Katowice, Medical University of Silesia, 40-635 Katowice, Poland; oag@poczta.onet.pl (O.G.-S.); kacper.kotyla@gmail.com (K.K.); jkurdybacha@gmail.com (J.K.); 2 Department of Rheumatology and Clinical Immunology, Voivodeship Hospital, No. 5, 41-200 Sosnowiec, Poland; 3 Health Promotion and Obesity Management Unit, Department of Pathophysiology, Faculty of Medical Sciences in Katowice, Medical University of Silesia, 40-752 Katowice, Poland; anna.m.chudek@gmail.com (A.C.); aowczarek@paintbox.pl (A.O.); 4 Department of Clinical Chemistry and Laboratory Diagnostics, Faculty of Pharmaceutical Sciences in Sosnowiec, Medical University of Silesia, 41-200 Katowice, Poland; kkuznik@sum.edu.pl (K.K.-T.); kvassev@sum.edu.pl (K.K.-V.); winsz@sum.edu.pl (K.W.-S.);; 5 Department of Internal Medicine and Oncological Chemotherapy, Medical University of Silesia, 40-029 Katowice, Poland; chj@poczta.fm

**Keywords:** systemic sclerosis, GDF15, galectin-3, MR-proANP, glutathione S-transferase π, mid-regional adrenomedullin, heart biomarkers, suPAR

## Abstract

Systemic sclerosis, a connective tissue disease of unknown etiology and unpredictable outcomes, is characterized by the fibrosis of the skin and internal organs, vasculopathy, and immune system dysregulation. The disease is classified into two main subtypes, which differ in clinical presentation, complications, and prognosis. While several biomarkers have been proposed to distinguish between these subtypes, none have achieved high sensitivity and specificity. The search for dependable markers that can differentiate between the two primary subtypes of systemic sclerosis continues. To address this gap, our study evaluated the utility of novel cardiac biomarkers, including growth differentiation factor 15 (GDF15), galectin-3, mid-regional pro-atrial natriuretic peptide (MR-proANP), glutathione S-transferase π, mid-regional adrenomedullin, and soluble urokinase plasminogen activator receptor (suPAR), in a cohort of 79 patients with both lcSSc and dSSc subtypes. The results demonstrated a significant elevation of GDF15 (medians: 2.07 vs. 1.10 ng/L; *p* < 0.001) and MR-proANP (92.55 vs. 65.60 pmol/L; *p* < 0.05) levels in SSc patients compared to healthy controls. Moreover, GDF15 (1.65 vs. 2.34 ng/mL; *p* < 0.05), MR-proANP (80.87 vs. 109.27 pmol/L; *p* < 0.05), and suPAR (1.83 vs. 2.44 ng/mL; *p* < 0.05) levels were notably higher in patients with dSSc compared to those with lcSSc. In the ROC analysis, only GDF-15, MR-proANP, and suPAR proved to have a statistically significant area under the curve (AUC). Patients with the GDF-15 ≥ 2182 ng/mL, MR-prANP ≥ 85.808 pmol/L, and suPAR ≥ 2.315 ng/mL have more than six-, eight-, and seven-times-higher odds for dcSSc, respectively. These findings highlight the potential of GDF15, suPAR, and MR-proANP as biomarkers for differentiating between the two main subtypes of systemic sclerosis.

## 1. Introduction

Systemic sclerosis is a connective tissue disease of unknown origin and unpredictable clinical course. It is characterized by skin and internal organ fibrosis, vasculopathy, and immune abnormalities, marked by the unequivocal presence of autoantibodies. While skin hardening and fibrosis constitute the hallmark of the disease, internal organ involvement and subsequent damage represent the most pressing concern in affected patients [1]. According to the canonical classification proposed by Le Roy, systemic sclerosis is categorized into two primary subtypes: limited and diffuse. The limited subtype is characterized by skin hardening that is restricted to the hands and feet, not extending beyond the level of the elbows and knees. In contrast, the diffuse subtype involves skin hardening that is also observable in the trunk, legs, and arms [2]. Distinguishing between the two main subtypes provides valuable clinical information regarding the disease’s progression, complications, and the spectrum of internal organ involvement. Currently, the distinction between subtypes is primarily based on the clinical presentation and the spectrum of autoantibodies found in individual patients. However, there are numerous cases where this classification is imprecise, leading to the inadequate categorization of many patients.

Considering the nearly 250 distinct metabolic and immunologic pathways through which inflammatory and profibrotic signals are mediated, biomarkers are likely to gain increasing attention in both research and clinical applications, particularly in the diagnosis and treatment of the disease. Presently, available biomarkers are predominantly associated with internal organ involvement and, to a lesser degree, with specific disease subtypes. However, a significant gap remains in the availability of biomarkers that can both differentiate disease subtypes and provide insights into cardiac function.

Recently, new biomarkers for heart dysfunction, such as growth differentiation factor 15 (GDF-15), galectin-3, mid-regional pro-atrial natriuretic peptide (MR-proANP), and soluble urokinase plasminogen activator receptor, have emerged. These biomarkers are extensively investigated in cardiovascular diseases, with particular emphasis on heart failure. Except for galectin-3, the role of these compounds in systemic sclerosis has not been assessed either comprehensively across the entire disease spectrum or specifically concerning the subtype of the disease.

The role of GDF-15 in the general population is being extensively investigated as a biomarker for both acute heart failure and the prediction of adverse outcomes. Elevated levels of this molecule are commonly observed in patients with heart failure both with preserved and reduced ejection fraction. However, the prognostic performance of GDF-15 in HFrEF and HFpEF is limited, as it is significantly influenced by renal function [3]. At the molecular level, GDF-15 is recognized as a biomarker reflecting oxidative stress, inflammation, and cellular aging [4]. In patients with systemic sclerosis, growth differentiation factor 15 (GDF-15) is associated with the diffuse subtype of the disease and its related complications.

These include arterial pulmonary hypertension, interstitial lung disease, and reduced forced vital capacity, as well as the presence of anti-TOPO I antibodies and an active capillaroscopic pattern observed during examination [5]. Furthermore, this study confirmed the role of GDF15 as a predictor for systemic sclerosis-related interstitial lung disease and elevated pulmonary artery pressure [6].

The mid-regional pro-atrial natriuretic peptide (MR-proANP) has been studied in patients with heart failure, with evidence suggesting its non-inferiority to NT-proBNP in diagnosing acute heart failure in the general population. In some studies, a significant value for risk stratification of adverse cardiovascular events in heart failure was demonstrated [7]. Furthermore, MR-proANP has proven to be a reliable marker for pulmonary hypertension in patients with systemic sclerosis [8]. However, to date, MR-proANP has not been extensively studied in patients with systemic sclerosis nor linked to any specific disease features. The same applies to mid-regional pro-adrenomedullin (MR-proADM), the peptide that demonstrated strong anti-inflammatory and anti-apoptotic effects. Similar to MR-proANP, MR-proADM has been recognized as a potential biomarker for pulmonary hypertension in patients with systemic sclerosis [8]. The peptide also plays a role in maintaining endothelial integrity, preserving microvascular circulation, reducing vascular resistance, and generally improving left ventricular function [9]. In several studies involving patients with heart failure, elevated levels of MR-proADM were associated with a worse cardiovascular prognosis [10,11]. However, the role of this peptide in systemic sclerosis has not been discussed, and data regarding its significance in SSc are lacking.

The final biomarker studied, suPAR, plays a crucial pathophysiological role by interacting with transmembrane proteins, such as integrins. This interaction is involved in the regulation of cell growth, migration, differentiation, and adhesion. suPAR mediates inflammation, the immune response, tissue remodeling, and angiogenesis, and is implicated in the progression of fibrotic diseases, such as systemic sclerosis (SSc) and rheumatoid arthritis [12]. There are limited reports on the role of soluble urokinase plasminogen activator receptor (suPAR) in systemic sclerosis (SSc), with studies generally indicating elevated suPAR levels in comparison to healthy controls. Given its significant profibrotic potential, it is plausible that suPAR may serve as an indicator of fibrotic and vascular manifestations, potentially acting as a valuable biomarker.

Moreover, a significant discrepancy is observed between the diffuse and limited forms of the disease, with suPAR levels being higher in the former [13,14].

A Plethora of Disease Biomarkers with Potential Utility in Systemic Sclerosis Has Been Proposed in Recent Years. However, None of Them Demonstrated Superiority Over NT-proBNP in Patients with Systemic Sclerosis

The use of NT-proBNP as a biomarker in systemic sclerosis (SSc), however, presents several limitations, including a lack of specificity. NT-proBNP serves mainly as an indicator of cardiac dysfunction and is influenced by comorbidities, such as pulmonary hypertension and chronic kidney disease. Furthermore, direct comparisons between BNP and MR-proANP in some studies have shown that MR-proANP is more strongly associated with the highest risk of adverse outcomes per proportional increase in plasma concentrations. It also demonstrated superior predictive value for recurrent heart failure, thereby diminishing the significance of BNP in multimarker prognostic models [7].

To address this gap, we evaluated the utility of several heart failure biomarkers in patients with systemic sclerosis, aiming to determine whether these biomarkers could effectively reflect cardiac status and aid in distinguishing disease subtypes. Furthermore, it aimed to correlate these biomarkers with disease progression, internal organ damage, and overall disease severity and activity.

## 2. Results

### 2.1. Study Group Characteristics

The observational cohort comprised 79 patients with scleroderma, with a mean age of 53.5 ± 12.0 years and a median disease duration of 5 years. The laboratory results were compared with those of 25 age- and sex-matched controls. Within the cohort, there was a predominance of patients with the diffuse type of the disease (51 vs. 28 patients), and the female-to-male ratio was 3:1. The disease duration from the first non-Raynaud symptom did not differ between the two groups of patients. All the patients were positive for antinuclear antibodies (ANA). A detailed analysis revealed that 49 (62.0%) patients were positive for Scl-70 antibodies, with 8 in the lcSSc and 41 in dcSSc. Additionally, anti-centromere antibodies (ACA) were present in 16 patients with lcSSc and 2 patients with dcSSc. The patients recruited for the study were characterized by high disease activity. Specifically, they exhibited widespread skin involvement with a mean modified Rodnan skin score (mRSS) of 12.1 (SD = 8.13) and a high European Scleroderma Study Group (EScSG) activity index of 3.58 (SD = 2.38). Additionally, with a disease duration exceeding five years, significant damage to internal organs and systems was observed, resulting in a high Medsger damage index, calculated for the entire group, with a mean value of 7.99 (SD = 3.58)—Table 1. The patients exhibited typical symptoms of the disease, including Raynaud’s phenomenon and skin damage, such as scars, active ulceration, telangiectasia, and digital necrosis. Esophageal involvement was observed in 70.1% of all the patients, defined as either persistent esophageal dysmotility or dilation of the esophagus on routine barium X-ray. Joint involvement was present in nearly 70% of the patients, identified through physical examination and assessments for joint edema or joint pain (arthralgia). However, no significant differences in the frequency of musculoskeletal system involvement were observed between the two patient groups. Given the nature of the disease, the physical examination of the joints also focused on tendon friction rubs, which were present in 36.7% of the patients, more prevalent although statistically insignificant in the diffuse type of the disease (25.0% vs. 43.1%; *p* = 0.18). Additionally, interstitial lung disease was diagnosed in 73.4% of the patients based on HRCT results, characterized by ground-glass opacities, subpleural reticulation with or without pleural irregularities, bronchiectasis, and honeycombing with pleural traction. More than half of the patients (52.6%) showed an active pattern in nailfold capillaroscopy, with an additional 11.8% patients displaying an early pattern. The patients received standard treatments for the disease, including cyclophosphamide, mycophenolate mofetil, azathioprine, and methotrexate. Some patients were treated with more than one immunosuppressant. Fourteen patients were treated with oral steroids, although the dose did not exceed 10 mg, as calculated based on prednisone. The complete clinical characteristics are presented in Table 1.

The analysis performed for the two disease subtypes revealed significant differences, presented in Table 2. Specifically, patients with limited cutaneous systemic sclerosis (lcSSc) exhibited lower modified Rodnan skin scores (mRSS 7.68 ± 4.93 vs. 14.53 ± 8.54; *p* < 0.001), had the less-active disease (EScSG 2.50 ± 1.66 vs. 4.16 ± 2.51; *p* < 0.01), and experienced less damage compared to patients with the diffuse form of scleroderma (Metsger 5.68 ± 2.55 vs. 9.25 ± 3.44; *p* < 0.001). Striking differences were also observed in the respiratory system: patients with dcSSc had a higher prevalence of interstitial lung disease, lower DLCO values, and poorer results in lung function tests (Table 2).

### 2.2. Heart Studies

Routine ECG records and historical data from patients’ charts revealed arrhythmias (17.7% vs. 54.9%; *p* < 0.05) and conduction disturbances (25.0% vs. 56.9%; *p* < 0.05) more frequently in the diffuse-type group. Routine echocardiographic studies did not show significant differences between the two disease groups in most of the parameters studied. The systolic function of the heart was preserved in all the subjects in both groups, although patients with lcSSc had slightly higher ejection fractions compared to those with the diffuse type (*p* < 0.01). Pulmonary hypertension was diagnosed in 17.1% of the entire group (data presented in Table 2). The frequency of this comorbidity was higher in the dcSSc group; however, the results did not reach statistical significance. Additionally, pulmonary hypertension was not correlated with the type of antinuclear antibody positivity or the presence of lung fibrosis. This finding may suggest the presence of type I arterial pulmonary hypertension, although invasive assessment was not performed.

### 2.3. Inflammatory and Heart Biomarkers

In general, patients with the diffuse type of systemic sclerosis were characterized by higher concentrations of inflammatory markers, reflecting a more severe disease course. Specifically, patients with dcSSc had elevated ESR 18 (12; 34) vs. 10 (5;16); *p* < 0.01) and CRP values above the upper normal limit (41.2% vs. 17.9%; *p* < 0.05). Additionally, beyond routine inflammatory markers, levels of two typical proinflammatory cytokines, TNF-α and IL-6, were assessed. Among these cytokines, only TNF-α levels were significantly elevated in patients compared to controls and were also higher in the diffuse systemic sclerosis (dcSSc) group compared to the limited systemic sclerosis group. No changes between lcSSc and dcSSC were noted in relation to NT-proBNP level. Several potentially new heart biomarkers were tested for their utility in systemic sclerosis patients. Among them, only GDF-15 (*p* < 0.001) and MR-proANP (*p* < 0.05) levels were found to be statistically higher in patients with systemic sclerosis compared to controls; more data emerged from a detailed analysis of heart biomarkers. When assessing the concentrations of biomarkers in dcSSc and lcSSc patients separately, we observed that MR-proANP (*p* < 0.05), GDF-15 (*p* < 0.05), and suPAR (*p* < 0.05) were elevated in patients with the diffuse type of the disease—Table 3.

In the second phase of the study, we performed linear regression or correlations to removedestablish the relationships between the biomarkers studied and various disease parameters. Among the biomarkers studied, GDF-15 correlated positively with markers of inflammation, such as CRP, leukocyte count, and platelet count, and strongly negatively with DLCO. Additionally, an association with leukocyte count was observed for both galectin-3 and MR-proADM, with the former showing a positive correlation (*p* = 0.36; *p* < 0.05) and the latter a negative one (*p* = −0.41; *p* < 0.01). Additional data come from the analysis of suPAR, which showed a negative correlation with DLCO (*p* = −0.41; *p* < 0.01). Finally, the biomarker GSTp failed to show any significant relationship with the parameters studied. No correlations were found between the biomarkers and disease activity, severity, damage, cardiac function (including ejection fraction and atrial fibrillation), or extent of skin involvement.

Furthermore, we assessed whether the inflammatory state, measured by the expression of typical proinflammatory cytokines (TNF-α and IL-6), could exert a regulatory effect on the expression of the cardiac biomarkers tested in this study. Our findings revealed that TNF-α positively correlated with the concentration of suPAR (ρ = 0.36; *p* < 0.05), while exerting a negative effect on MR-proADM levels (ρ = −0.38; *p* < 0.05). Furthermore, a significant positive correlation was observed between galectin-3 and suPAR concentrations (ρ = 0.40; *p* < 0.001).

We also checked whether biomarkers can distinguish between the diffuse subtype and limited type and if it is possible to find a cut-off of biomarkers dividing both subtypes. In the ROC analysis, only GDF-15, MR-proANP, and suPAR proved to have a statistically significant area under the curve (AUC). The most sensitive (Se) biomarker for distinguishing dSSc from IsSSc was MR-proANP, which also has the highest value of negative predictive value (NPV). The GDF-15 proved to be the most specific (Sp) biomarker with the highest value of positive predictive value (PPV-) as presented in Table 4.

**Figure 1:**
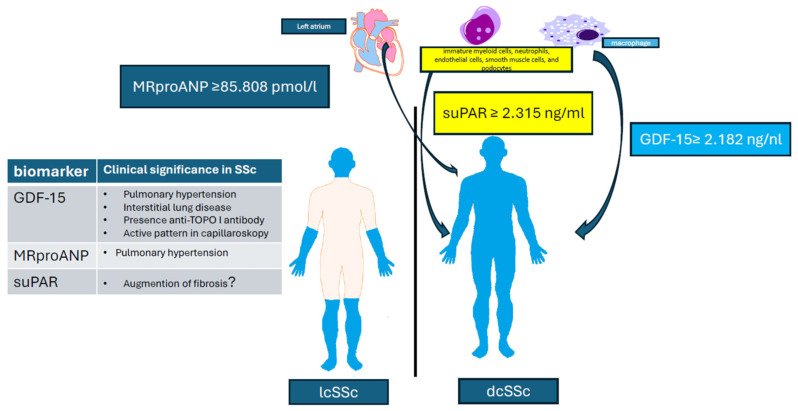
Role of Biomolecules in Two Subtypes of Systemic Sclerosis.

Patients with the GDF-15 value ≥2.182 ng/mL have more than 6-times-higher odds for dSSc (OR = 6.46, 95% CI: 1.54–27.18; *p* < 0.01), with the MR-proANP value ≥85.808 pmol/L have more than 8-times-higher odds for dcSSc (OR = 8.25, 95% CI: 2.05–33.16; *p* < 0.01), and with the suPAR value ≥2.315 ng/mL have more than 7-times-higher odds for dcSSc (OR = 7.48, 95% CI: 1.74–32.18; *p* < 0.01). Taking all the results into account, we can say that MR-proANP is the best biomarker distinguishing both types of SSc—Figure 1.

Three biomolecules proposed as biomarkers for differentiating between diffuse and limited systemic sclerosis are produced by progenitor myelopoietic cells (SuPAR), the muscle of the left atrium of the heart (MR-proANP), and macrophages (GDF-15). In systemic sclerosis, these molecules are associated with various disease complications, including pulmonary hypertension, lung fibrosis, and microvascular damage (evidenced by an active pattern in capillaroscopy). In contrast to their use in the general population as biomarkers of heart function and heart failure, these molecules are not associated with heart function in systemic sclerosis.

## 3. Discussion

The search for new biomarkers in a given disease is crucial, particularly in light of the disease’s heterogeneity. This task is especially challenging in systemic sclerosis due to its complex clinical presentation. An ideal biomarker should correlate with the clinical parameters of the disease, aiding physicians in making accurate diagnoses and drawing appropriate conclusions that lead to effective therapeutic interventions. In recent years, several novel biomarkers for cardiac function have been proposed. However, except for galectin-3, and to a lesser degree suPAR, the utility of these molecules has not yet been extensively tested in systemic sclerosis [14]. We excluded from this study patients with overlap syndrome, diagnosed malignancies, and a history of prior cardiovascular events. This was done to preserve the homogeneity of the patient cohort, considering that biomarker levels may be influenced by various comorbidities, particularly other connective tissue diseases, cancer, and advanced atherosclerosis. By doing so, we aimed to assess the influence of the primary disease on the studied parameters. In our study, we observed no significant difference in galectin-3 levels between patients with systemic sclerosis and the control group, nor between those with a limited cutaneous and diffuse subtype of the disease. This finding contrasts with previous research and may be attributable to the inclusion of patients with longer disease durations This observation aligns with the work of Taniguchi et al., who demonstrated that disease duration has a significant impact on galectin-3 levels [15]. Specifically, galectin-3 levels are significantly reduced in the early stages of the disease but tend to increase as the disease progresses. Furthermore, the results of our study stand in stark contrast to those of Sundblad et al., an Argentinian group that reported differences in galectin-3 levels between the diffuse and limited subtypes of systemic sclerosis and established a relationship between galectin-3 levels and clinical presentation [16]. The discrepancies between these studies may be attributed to differences in patient populations. In Sundblad’s study, the dcSSc to lcSSc ratio was 1:3, whereas, in our study, the ratio was 3:1 in favor of the diffuse subtype. Additionally, Sundblad’s study included patients with overlap syndromes, whereas our study excluded such patients. These differences might also account for the lack of correlation between galectin-3 concentration and clinical features in our study. To our surprise, we did not observe any relationship between cardiac function (including pulmonary hypertension, left ventricular systolic function, and NT-proBNP levels) and the concentration of galectin-3, despite galectin-3 being widely recognized as a promising biomarker not only in systemic sclerosis patients but also in the general population with cardiovascular diseases [17,18,19].

More promising findings emerged from the analysis of other biomarkers, particularly GDF15. In our study, GDF15 levels were significantly higher in patients with systemic sclerosis compared to healthy controls and also varied significantly between disease subtypes, consistent with previous reports. Notably, our results align with the study by Oller-Rodrigues, which also observed striking differences in GDF15 levels between lcSSc and dcSSc [5]. Furthermore, GDF15 was associated with hematopoietic system function and lung function (DLCO). Regarding the latter, our findings are in perfect agreement with those of Meadows et al., who reported an inverse correlation between GDF15 and DLCO [20]. However, unlike Meadows’ study, we did not find any relationship between GDF15 levels and pulmonary hypertension or NT-proBNP levels. The confirmation of previous findings on GDF-15 supports its potential as a promising biomarker. However, given the discrepancies observed between studies, further research is necessary to clarify the precise role of this biomarker. Considering the variations in disease duration and patient characteristics between our study and those previously published, it is crucial to determine the appropriate stage of systemic sclerosis during which GDF-15 should be assessed. This approach appears reasonable in light of a five-year follow-up study conducted in the Czech Republic, where GDF-15 levels were found to increase significantly over time in conjunction with disease progression [21]. Additionally, it is important to identify the specific disease subtypes in which measuring GDF-15 concentrations may provide valuable clinical insights.

This study represents the first instance in which the soluble urokinase plasminogen activator receptor (suPAR) has been evaluated as a potential biomarker for both heart function and the subtype of systemic sclerosis. Although suPAR levels did not significantly differ between patients and controls, they were statistically higher in patients with the diffuse subtype of the disease. It appears plausible that suPAR is positively regulated by TNF-α in systemic sclerosis, as the levels of these molecules are mutually correlated. Furthermore, a detailed correlation analysis revealed that suPAR expression is associated with clinical manifestations of systemic sclerosis, such as lung function (DLCO) and NT-proBNP levels. Our findings are consistent with previous studies, which demonstrated a positive correlation between suPAR levels and DLCO in patients with systemic sclerosis [13]. In the general population, elevated suPAR levels are indicative of low-grade inflammation, aligning with the regulation of suPAR by TNF-α observed in our study [22]. Furthermore, as established in the general population, suPAR levels are significantly correlated with various inflammatory markers, including TNF-α, which our findings also corroborate [23]. Therefore, it is unsurprising that higher suPAR levels were observed in patients with diffuse cutaneous systemic sclerosis (dcSSc), a more severe form of the disease. Collectively, these results reinforce the role of suPAR as a potential biomarker in systemic sclerosis. However, its correlation with only a single cardiovascular marker, NT-proBNP, suggests a very limited utility of suPAR in assessing cardiac function. The role of suPAR in predicting new-onset atrial fibrillation remains highly controversial, with one study suggesting a potential association [24] and another failing to confirm it [25]. Consequently, the higher prevalence of atrial fibrillation observed in patients with the diffuse form of the disease is likely to have little or no influence on the concentration of this biomarker. Another potential biomarker for systemic sclerosis investigated in our study was the mid-regional pro-atrial natriuretic peptide (MR-proANP), a member of the large natriuretic peptide family. The significance of this biomarker has been increasingly recognized, as its measurement offers valuable insights into the cardiovascular status of patients, particularly in conditions such as pulmonary hypertension, heart failure, or pulmonary embolism [26,27,28]. Only one study has investigated MR-proANP levels in systemic sclerosis, and it was limited to a cohort of patients with pulmonary hypertension [8]. Even though mid-regional pro-atrial natriuretic peptide (MR-proANP) is a well-established prognostic marker in various inflammatory, respiratory, and cardiovascular conditions, its role in systemic sclerosis, particularly concerning disease activity, damage, and progression, has not yet been thoroughly explored. In our study, MR-pro ANP levels were found to be elevated in patients with SSc compared to healthy controls. Additionally, MR-pro ANP was effective in distinguishing between patients with limited cutaneous systemic sclerosis (lcSSc) and diffuse cutaneous systemic sclerosis (dcSSc), with significantly higher levels observed in the latter group. Moreover, the ROC analysis demonstrated that MR-pro ANP exhibited the greatest accuracy as a biomarker for differentiating between the two subtypes of the disease. Our findings suggest that MR-pro ANP may serve as a promising biomarker for systemic sclerosis and its subtypes. Unexpectedly, we were unable to demonstrate any association between cardiac function, specifically cardiac arrhythmias such as atrial fibrillation (AF), and the biomarkers evaluated in our study. This finding is particularly surprising given the higher incidence of atrial fibrillation reported in patients with the diffuse form of the disease.

In the general population, changes in mid-regional pro-atrial natriuretic peptide (MR-proANP) levels are commonly utilized to identify individuals at elevated risk for developing AF, cardioembolic stroke, and subsequent cardiovascular events following stroke [29,30]. However, the relationship between already-established AF and MR-proANP levels remains less well understood. Consequently, it is unclear whether the association between MR-proANP and the onset of AF is specific to ischemic stroke or may be generalized to all instances of atrial fibrillation. Moreover, in the general population, elevated MR-proANP levels have been linked to inflammatory and septic conditions, suggesting a potential regulatory role of inflammatory cytokines — particularly TNF-α — in the expression of this biomarker, a mechanism also supported by findings in our study [31]. There is limited knowledge regarding the role of MR-proADM and GSTp in systemic sclerosis. MR-proADM has been studied in relation to pulmonary hypertension in systemic sclerosis patients, with conflicting results reported [8,32]. However, in our study, we did not find any significant relationship between MR-proADM and systemic sclerosis, the clinical presentation of the disease, or its subtypes. This may be attributed to the relatively small sample size, as only 14 patients (17.7%) were diagnosed with pulmonary hypertension. Additionally, we relied solely on echocardiographic examination, which is less precise than the gold standard of right heart catheterization for the diagnosis of pulmonary hypertension. It is also plausible to suggest, in line with the findings of the study by ten Freyhaus et al., that MR-proADM may not have a significant role in the assessment of pulmonary hypertension associated with systemic sclerosis [32].

The same holds for GSTp levels. In our study, the levels of this biomolecule did not differ between patients and controls, nor between the two subtypes of the disease. To date, GSTp levels have been assessed in the bronchoalveolar fluid of systemic sclerosis patients, where they were found to be downregulated in individuals with lung fibrosis and were thought to play a protective role in the development of pulmonary fibrosis [33]. Despite GSTp being proposed as a valuable marker for predicting ventricular function in heart failure patients, we did not observe any relationship between its levels and cardiac function or the clinical presentation of the disease in our study [34].

### Study Limitations

This study has several limitations. While we aimed to recruit a relatively large cohort of patients, as a single-center study, the sample size, though substantial, may still be insufficient to detect subtle associations that might emerge in larger populations. We focused primarily on left ventricular systolic function, potentially overlooking patients with heart failure with preserved ejection fraction (HFpEF), which is the predominant form of cardiac involvement in this disease. Pulmonary hypertension was classified based solely on echocardiography, which may lead to the misclassification of some cases. Consequently, conclusions regarding the relationship between biomarkers and heart function should be interpreted with caution, as they reflect only systolic heart function. Additionally, we did not perform right heart catheterization, which may have led to the underdiagnosis or overdiagnosis of pulmonary hypertension, as some cases would require catheterization for definitive confirmation. Finally, the results of our study should be interpreted with caution, as it is not possible to entirely exclude the influence of inflammatory status on the biomarkers studied. In this context, we observed the impact of TNF-alpha on suPAR and MR-proADM levels, as well as a reciprocal relationship between galectin-3 and suPAR. Thus, we cannot definitively rule out the potential involvement of other proinflammatory cytokines and the broader inflammatory status. This limitation may affect the utility of the biomarkers examined but underscores the need for further research in this area.

## 4. Material and Methods

In this cross-sectional study, we recruited 79 patients diagnosed with systemic sclerosis based on the ACR/EULAR criteria. Patients were recruited utilizing the local systemic sclerosis database of the Department of Rheumatology and Clinical Immunology in Voivodeship Hospital No 5 in Sosnowiec. According to the criteria proposed by Le Roy, we further characterized patients based on the extent of skin involvement, categorizing them into lcSSc and dcSSc. The disease duration has been calculated from the first non-Raynaud symptom attributable to SSc. We excluded patients previously or currently treated with biologics (TNF inhibitors, IL-6 antagonists, and co-stimulation inhibitors) and those receiving B cell depletion therapy, suffering from overlap syndromes (including but not limited to SSc/SLE, SSc/PM, and SSc/RA), cancer, and those who had undergone bone marrow transplantation or experienced cardiovascular events (myocardial infarction or drug-related cardiotoxicity), as well as pregnant and lactating women.

The patients recruited for the study underwent detailed physical examinations and comprehensive medical history assessments, which included demographic data, comorbidities, disease manifestations, and concurrent treatments. Standard laboratory assessments were conducted in all the subjects, encompassing haematological parameters, liver function tests, and the quantification of creatinine levels. The presence of antinuclear antibodies (ANAs) was screened using the indirect immunofluorescence method with Hep-2 cell lines. Subsequently, specific antibodies were further analyzed using an enzyme-linked immunosorbent assay (ELISA), including antibodies targeting topoisomerase I (Scl-70 Topo I), anticentromere, U1RNP, U3RNP, polymerase I and III, Th/Th0, Ro, La, and Ro52. (Euroimmun Lübeck, Groß Grönau, Germany).

The extent of skin fibrosis was measured using the modified Rodnan skin score across 17 specific regions, assessed by the same experienced examiner to minimize interobserver bias. Esophageal dysmotility was evaluated based on the results of barium X-rays. Clinical presentations such as arthritis, tenosynovitis, fingertip deformities, ulcerations, telangiectases, and tendon friction rubs were assessed by the same experienced rheumatologist, blinded to the biomarker assessment results. Per institutional policy, all subjects with systemic sclerosis underwent routine high-resolution computed tomography (HRCT) at the time of diagnosis and were re-evaluated within 1–2 years, or earlier if necessary. Consequently, all the patients had an HRCT examination no older than 12 months. Based on HRCT results, lung fibrosis was formally identified, and lung abnormalities were further categorized into typical features such as ground-glass opacities and honeycombing. Additionally, all the patients underwent lung function tests within two weeks before the study assessment. These tests included measurements of forced vital capacity (FVC), the ratio of forced expiratory volume in one second to forced vital capacity (FEV1/FVC), total lung capacity (TLC), and single-breath diffusing capacity for carbon monoxide corrected for hemoglobin (DLCO), conducted following the guidelines of the American Thoracic Society/European Respiratory Society. The results were expressed as percentages of predicted values. Nailfold capillaroscopy was performed according to standard procedures using a Dino-Lite capillaroscope. (Dino-Lite Europe NN Almere, The Netherlands)

Heart assessments were conducted clinically through heart and chest examinations, followed by resting ECG recording, and echocardiography. Transthoracic echocardiographic and Doppler examinations were performed by an experienced cardiologist who was blinded to the clinical data. The calculation of left ventricular ejection fraction was carried out using the modified Simpson’s rule, as per the built-in software. Pulmonary arterial hypertension (PAH) was diagnosed in patients who demonstrated a tricuspid regurgitant jet velocity greater than 2.8 m/s, along with additional echocardiographic indicators suggestive of PAH, following the 2022 guidelines of the European Society of Cardiology (ESC) and the European Respiratory Society (ERS) [35].

The patients were receiving a variety of treatment regimens, which included the use of immunosuppressants. The eligible-for-the-study patients were on a stable treatment regimen for at least three months. Steroid use was permitted at doses not exceeding 10 mg/day, while immunosuppressants were allowed at standard doses (methotrexate < 25 mg/week; mycophenolate mofetil < 2.0 g/day; and azathioprine < 200 mg/day). Intravenous cyclophosphamide was also permitted at cumulative doses not exceeding 1000 mg/month, provided it had been administered at a stable dose for at least three months for interstitial lung disease. To minimize the drug-related bias, an assessment of heart dysfunction biomarkers was performed no earlier than 28 days after the last infusion.

Patients were required to discontinue vasodilators, including calcium channel blockers and angiotensin-converting enzyme inhibitors, at least three days before inclusion to ensure more than five times the drug’s half-life had elapsed. Disease characteristics were thoroughly documented, including disease-related damage, assessed using the Medsger scale [36], and disease activity, measured according to the 2017 EUSTAR Activity Index [37]. A group of 25 age- and sex-matched individuals served as controls.

This study was designed and conducted in compliance with the Declaration of Helsinki, and the protocol was approved by the Bioethical Committee of the Medical University of Silesia, Poland (document identification code: PCN/0022/KB1/17/I/20/21, issued on 30 March 2021). Informed consent was obtained from all the participants before the initiation of any study procedures.

### 4.1. Laboratory Analysis

Peripheral blood samples were collected on ethylenediaminetetraacetic acid (EDTA) as an anticoagulant from the forearm in the morning alongside routine analyses typically performed in hospitalized individuals. The serum concentrations of cardiac biomarkers were determined using commercially available ELISA assays as follows: GDF15, (BioVendor, Brno, the Czech Republic); MR-proANP (Cusabio, Wuhan, China; galectin-3 (BioVendor, Brno, the Czech Republic); IL-6 (BioVendor, Brno, the Czech Republic); glutathione S-transferase π-GSTP (Immunodiagnostic, Bensheim, Germany); MR-proADM (Abbexa, Cambridge, UK); and suPAR (BioVendor, Brno, the Czech Republic).

### 4.2. Statistical Analysis

Statistical analysis was performed using STATISTICA 13.0 PL (TIBCO Software Inc., Palo Alto, CA, USA) and R software v. 4.4.0 [R Core Team (2013). R: A language and environment for statistical computing. R Foundation for Statistical Computing, Vienna, Austria. http://www.R-project.org/, accessed 24 April 2024]. Statistical significance was set at a *p*-value below 0.05. All the tests were two-tailed. Imputations were not conducted for missing data. No multiple comparison methods were used in statistical analysis. Nominal and ordinal data were expressed as percentages. Interval data were expressed as the median, with lower (Q_1_) and upper (Q_3_) quartiles, or the mean with standard deviation (SD), depending on the data distribution. The distribution of variables was evaluated by the Anderson–Darling test and the quantile–quantile (Q–Q) plot. The homogeneity of variances was assessed by the Levene test. Comparisons between the two groups were performed with either the Mann–Whitney U test or with χ^2^ tests. To find the cut-off distinguishing diffuse and limited SS, the ROC curve analysis with the Youden index was used. The results were presented as the area under the curve (AUC), sensitivity (Se), specificity (Sp), and positive and negative predictive values (PPN and NPV, respectively). The Spearman rank correlation coefficient was used as a measure of association between variables.

## 5. Conclusions

A diverse range of novel cardiac biomarkers is continually being proposed for predicting cardiac function across various diseases. However, as demonstrated in our study, even well-established biomarkers that are effective in the general population may not perform equally well in the specific context of systemic sclerosis. Nevertheless, our findings identify three cardiac biomarkers that could assist in identifying patients with systemic sclerosis and differentiating between its two subtypes. These findings, if replicated in other studies, particularly multicenter trials, could be of significant importance. They may help distinguish between the two main subtypes of the disease and provide further insights into its course, complications, and outcomes.

Notably, MR-proANP, with its high capacity to distinguish between the two subtypes of the disease, emerges as a particularly promising biomarker.

## Figures and Tables

**Table 1:** Demographic and disease activity characteristics of the study cohort.

Parameter	
Gender, *n* (%)	Female:male 59:20 (74.7%:25.3%)
Age (years), mean ± SD	53.5 ± 12.0
Disease duration (from non-Raynaud symptoms) (Years), median (Q1;Q3)	5.0 (2.0; 9.0)
Disease subtype, *n* (%)	Limited 28 (35.4%) Diffuse 51 (64.6%)
Serological status, *n* (%) Anti-SCL70 Anti-ACA Anti-RNP	49 (62%) 18 (22.8%) 2 (2.5%)
Capilaroscopic pattern, *n*(%) Early Active Late Normal/other	9 (11.8%) 40 (52.6%) 25 (32.9%) 4 (5.3%)
Activity/Severity	
Skin involvement (mRSS) [pts], mean ±SD	12.10 ± 8.13
Disease severity/damage (Medsger scale) [pts], mean ± SD	7.99 ± 3.58
Disease activity (European Scleroderma Study Group (EScSG) Activity Index) [pts], mean ± SD	3.58 ± 2.38
ESR (mm/h), median (Q1;Q3)	14 (8; 26)
DLCO [% expected value], mean ± SD	73.2 ± 20.3
NT-proBNP [ng/L] Median (Q1;Q3)	169.0 (89.0; 396.0)

Anti-SCL70 antibody against SCL 70 (topoisomerase I); Anti-ACA—anticentromere antibody, Anti-RNP a—antibodies to ribonucleoprotein; mRSS—modified Rodnan Skin Score; ESR—erythrocyte sedimentation rate, DLCO—diffusing capacity of the lungs for carbon monoxide; NT-proBNP— N-terminal pro b-type natriuretic peptide.

**Table 2:** Clinical Characteristics of the Patients.

Clinical Characteristics	All	Limited (lcSSc)	Diffuse (dcSSc)	*p* Value *
Raynaud phenomenon, *n* (%)	77 (97.5%)	27 (96.4%)	50 (98.0%)	>0.99
Proximal muscle weakness, *n* (%)	33 (41.8%)	10 (35.7%)	23 (45.1%)	0.57
Tendon friction rubs, *n* (%)	29 (36.7%)	7 (25.0%)	22 (43.1%)	0.18
Gastrointestinal involvement, *n* (%)	56 (70.9%)	16 (57.1%)	40 (78.4%)	0.08
Interstitial lung disease, *n* (%)	58 (73.4%)	13 (46.4%)	45 (88.2%)	<0.001
Pulmonary hypertension, *n* (%)	14 (17.7%)	3 (10.7%)	11 (21.6%)	0.36
Coronary artery disease, *n* (%)	28 (35.4%)	7 (25.0%)	21 (41.2%)	0.23
ECG conduction disturbances, *n* (%)	36 (45.6)	7 (25.0%)	29 (56.9%)	<0.05
ECG arytmia, *n* (%) Atrial fibrillaton, *n* (%) Premature ventricular complexes	35 (44.3%) 12 (15.1%) 15 (18.9%)	5 (17.9%) 2 (2.5%) 7 (8.8%)	28 (54.9%) 10 (12.6%) 8 (10.1%)	<0.01 <0.05 0.21
Echocardiographic parameters				
(LA) [mm]	35.1 ± 5.0	34.8 ± 6.0	37.4 ± 5.4	0.17
LVEDd [mm]	49.3 ± 2.9	48.5 ± 4.1	49.7 ± 6.5	0.18
LVESd [mm]	29.2 ± 4.4	29.0 ± 3.0	30.6 ± 7.6	0.26
Ao [mm]	33.6 ± 3.3	32.6 ± 3.4	35 ± 4.1	0.12
RVEDd (RV) [mm]	26.6 ± 3.6	26.3 ± 3.5	28.1 ± 4.5	0.24
IVSd [mm]	10.1 ± 1.1	9.7 ± 1.4	10.5 ± 1.4	0.33
PWTd (LVPW) [mm]	9.8 ± 0.8	9.6 ± 0.7	10.1 ± 1.1	0.59
Ejection fraction, Median (25%; 75%)	60.00 (55.00; 60.00)	60.00 (60.00; 60.00)	60.00 (55.00; 60.00)	<0.01
Dyspnoë, *n* (%)	44 (55.7%)	8 (28.6%)	36 (70.6%)	<0.001
Hypocomplementemia, *n* (%)	9 (11.3%)	3 (10.7%)	6 (11.8%)	>0.99
Ground glass HRCT, *n* (%)	40 (50.6%)	9 (32.1%)	31 (60.8%)	<0.05
Puffy fingers current, *n* (%)	37 (46.8)	12 (42.9%)	25 (49.0%)	0.77
Scleroderma capillary pattern early, *n* (%)	9 (11.3%)	3 (11.1%)	6 (12.2%)	>0.99
Scleroderma capillary pattern active, *n* (%)	40 (50.6%)	15 (55.6%)	25 (51.0%)	0.89
Scleroderma capillary pattern late, *n* (%)	25 (31.6%)	8 (29.6%)	17 (34.7%)	0.85
Non-specific capillary pattern, *n* (%)	4 (5.0%)	3 (11.1%)	1 (2.0%)	0.25
Dry cough, *n* (%)	36 (45.6)	10 (35.7%)	26 (51.0%)	0.29
Muscle_pain, *n* (%)	36 (45.6)	11 (39.3%)	25 (49.0%)	0.55
Arthritis, *n* (%)	55 (69.6)	20 (71.4%)	35 (68.6%)	>0.99

* Comparison between diffuse and limited type of the disease, LA left atrium, LVEDd—left ventricular end-diastolic diameter, LVESd—left ventricular end-systolic diameter, Ao—aorta, RVEDd right ventricular end-diastolic diameter, IVSd—interventricular septum diameter, and PWTd (LVPW)—posterior wall thickness.

**Table 3:** Comparison of heart biomarkers and proinflammatory cytokines profile in patients with systemic sclerosis and healthy controls according to the subtype of the disease.

Parameter	Patients, *n* = 79	Controls, *n* = 27	*p* Value ^1^	Limited SSc, *n* = 28	Diffuse SSc, *n* = 51	*p* Value ^2^
GDF15 (ng/mL)	2.07 (1.18; 2.93)	1.10 (0.82; 1.24)	<0.001	1.65 (0.82; 2.02)	2.34 (1.36; 3.06)	<0.05
Galectin 3 (ng/mL)	0.45 (0.09; 1.54)	0.22 (0.08; 0.87)	0.40	0.16 (0.09; 0.73)	0.56 (0.08; 1.90)	0.46
GSTp (ng/mL)	0.23 (0.12; 0.51)	0.27 (0.13; 0.96)	0.38	0.24 (0.16; 0.30)	0.23 (0.12; 0.56)	0.94
MR-proANP (pmol/L)	92.55 (76.52; 166.61)	65.60 (56.85; 129.11)	<0.05	80.87 (65.59; 86.80)	109.27 (82.41; 195.22)	<0.05
suPAR (ng/mL)	2.19 (1.77; 2.80)	1.94 (1.69; 2.25)	0.21	1.83 (1.75; 2.19)	2.44 (1.91; 2.93)	<0.05
MR-proADM (pmol/L)	1.75 (1.25; 2.64)	1.71 (1.30; 1.82)	0.30	1.98 (1.17; 2.98)	1.71 (1.25; 1.97)	0.40
TNF-α (pg/mL)	5.63 (3.85; 8.64)	10.50 (6.64; 21.50)	<0.01	4.41 (3.53; 5.27)	6.82 (4.46; 8.93)	<0.05
IL-6 (pg/mL)	8.85 (2.00; 48.99)	2.30 (2.00; 35.60)	0.72	2.00 (2.00; 28.38)	13.05 (2.00; 52.11)	0.31

Median (Q_1_; Q_3_), ***p* Value ^1^ patients compare to controls, *p* Value ^2^** patients with dcSSc compared to patients with lcSSc.

**Table 4:** Results of the ROC analysis.

Biomarker	Cut-Off	AUC	*p*	Se (%)	Sp (%)	PPV (%)	NPV (%)
GDF-15 (ng/mL)	≥2.182	0.706 (0.541–0.870)	<0.05	61.3 (42.2–78.2)	82.0 (56.6–96.2)	86.4 (63.9–93.5)	53.8 (35.0–84.6)
MR-proANP (pmol/L)	≥85.808	0.714 (0.558–0.871)	<0.01	73.3 (54.1–87.7)	75.0 (47.6–92.7)	84.6 (62.5–93.5)	60.0 (39.1–86.5)
suPAR (ng/mL)	≥2.315	0.700 (0.543–0.857)	<0.05	63.3 (43.9–80.1)	81.2 (54.4–96.0)	86.4 (63.5–93.6)	54.2 (34.8–86.6)

Mean value (95% confidence interval); AUC—area under the curve, Se—sensitivity, Sp—specificity, and PPV/NPV—positive/negative predictive value.

## Data Availability

The datasets analyzed or generated during the study are available from the corresponding author.

## References

[1] Allanore Y., Simms R., Distler O., Trojanowska M., Pope J., Denton C.P., Varga J. (2015). Systemic sclerosis. Nat. Rev. Dis. Primers.

[2] LeRoy E.C., Black C., Fleischmajer R., Jablonska S., Krieg T., Medsger T.A., Rowell N., Wollheim F. (1988). Scleroderma (systemic sclerosis): Classification, subsets and pathogenesis. J. Rheumatol..

[3] Castiglione V., Aimo A., Vergaro G., Saccaro L., Passino C., Emdin M. (2022). Biomarkers for the diagnosis and management of heart failure. Heart Fail. Rev..

[4] di Candia A.M., de Avila D.X., Moreira G.R., Villacorta H., Maisel A.S. (2021). Growth differentiation factor-15, a novel systemic biomarker of oxidative stress, inflammation, and cellular aging: Potential role in cardiovascular diseases. Am. Heart J. Plus Cardiol. Res. Pract..

[5] Oller-Rodríguez J.E., Vicens Bernabeu E., Gonzalez-Mazarío R., Grau García E., Ortiz Sanjuan F.M., Román Ivorra J.A. (2022). Utility of cytokines CXCL4, CXCL8 and GDF15 as biomarkers in systemic sclerosis. Med. Clin..

[6] Kawashiri S.Y., Nishino A., Igawa T., Takatani A., Shimizu T., Umeda M., Fukui S., Okada A., Suzuki T., Koga T. (2018). Prediction of organ involvement in systemic sclerosis by serum biomarkers and peripheral endothelial function. Clin. Exp. Rheumatol..

[7] Tan E.S.J., Chan S.P., Liew O.W., Chong J.P.C., Gerard Leong K.T., Daniel Yeo P.S., Ong H.Y., Jaufeerally F., Sim D., Ling L.H. (2024). Differential Associations of A-/B-Type Natriuretic Peptides With Cardiac Structure, Function, and Prognosis in Heart Failure. JACC Heart Fail..

[8] Miller L., Chartrand S., Koenig M., Goulet J.R., Rich É., Chin A.S., Chartrand-Lefebvre C., Abrahamowicz M., Senécal J.L., Grodzicky T. (2014). Left heart disease: A frequent cause of early pulmonary hypertension in systemic sclerosis, unrelated to elevated NT-proBNP levels or overt cardiac fibrosis but associated with increased levels of MR-proANP and MR-proADM: Retrospective analysis of a French Canadian cohort. Scand. J. Rheumatol..

[9] Eto T. (2001). A review of the biological properties and clinical implications of adrenomedullin and proadrenomedullin N-terminal 20 peptide (PAMP), hypotensive and vasodilating peptides. Peptides.

[10] Caruhel P., Mazier C., Kunde J., Morgenthaler N.G., Darbouret B. (2009). Homogeneous time-resolved fluoroimmunoassay for the measurement of midregional proadrenomedullin in plasma on the fully automated system B.R.A.H.M.S KRYPTOR. Clin. Biochem..

[11] Neumann J.T., Tzikas S., Funke-Kaiser A., Wilde S., Appelbaum S., Keller T., Ojeda-Echevarria F., Zeller T., Zwiener I., Sinning C.R. (2013). Association of MR-proadrenomedullin with cardiovascular risk factors and subclinical cardiovascular disease. Atherosclerosis.

[12] Kanno Y. (2023). The uPA/uPAR System Orchestrates the Inflammatory Response, Vascular Homeostasis, and Immune System in Fibrosis Progression. Int. J. Mol. Sci..

[13] Butt S., Jeppesen J.L., Iversen L.V., Fenger M., Eugen-Olsen J., Andersson C., Jacobsen S. (2021). Association of soluble urokinase plasminogen activator receptor levels with fibrotic and vascular manifestations in systemic sclerosis. PLoS ONE.

[14] Legány N., Toldi G., Distler J.H., Beyer C., Szalay B., Kovács L., Vásárhelyi B., Balog A. (2015). Increased plasma soluble urokinase plasminogen activator receptor levels in systemic sclerosis: Possible association with microvascular abnormalities and extent of fibrosis. Clin. Chem. Lab. Med..

[15] Taniguchi T., Asano Y., Akamata K., Noda S., Masui Y., Yamada D., Takahashi T., Ichimura Y., Toyama T., Tamaki Z. (2012). Serum Levels of Galectin-3: Possible Association with Fibrosis, Aberrant Angiogenesis, and Immune Activation in Patients with Systemic Sclerosis. J. Rheumatol..

[16] Sundblad V., Gomez R.A., Stupirski J.C., Hockl P.F., Pino M.S., Laborde H., Rabinovich G.A. (2021). Circulating Galectin-1 and Galectin-3 in Sera From Patients With Systemic Sclerosis: Associations With Clinical Features and Treatment. Front. Pharmacol..

[17] Fenster B.E., Lasalvia L., Schroeder J.D., Smyser J., Silveira L.J., Buckner J.K., Brown K.K. (2016). Galectin-3 levels are associated with right ventricular functional and morphologic changes in pulmonary arterial hypertension. Heart Vessels.

[18] Shah R.V., Chen-Tournoux A.A., Picard M.H., van Kimmenade R.R., Januzzi J.L. (2010). Galectin-3, cardiac structure and function, and long-term mortality in patients with acutely decompensated heart failure. Eur. J. Heart Fail..

[19] Vértes V., Porpáczy A., Nógrádi Á., Tőkés-Füzesi M., Hajdu M., Czirják L., Komócsi A., Faludi R. (2022). Galectin-3 and sST2: Associations to the echocardiographic markers of the myocardial mechanics in systemic sclerosis—A pilot study. Cardiovasc. Ultrasound.

[20] Meadows C.A., Risbano M.G., Zhang L., Geraci M.W., Tuder R.M., Collier D.H., Bull T.M. (2011). Increased expression of growth differentiation factor-15 in systemic sclerosis-associated pulmonary arterial hypertension. Chest.

[21] Hromadka M., Baxa J., Seidlerova J., Miklik R., Rajdl D., Sudova V., Suchy D., Rokyta R. (2021). Myocardial Involvement Detected Using Cardiac Magnetic Resonance Imaging in Patients with Systemic Sclerosis: A Prospective Observational Study. J. Clin. Med..

[22] Rasmussen L.J.H., Petersen J.E.V., Eugen-Olsen J. (2021). Soluble Urokinase Plasminogen Activator Receptor (suPAR) as a Biomarker of Systemic Chronic Inflammation. Front. Immunol..

[23] Koch A., Voigt S., Kruschinski C., Sanson E., Dückers H., Horn A., Yagmur E., Zimmermann H., Trautwein C., Tacke F. (2011). Circulating soluble urokinase plasminogen activator receptor is stably elevated during the first week of treatment in the intensive care unit and predicts mortality in critically ill patients. Crit. Care.

[24] Westin O., Rasmussen L., Andersen O., Buch E., Eugen-Olsen J., Friberg J. (2018). Soluble Urokinase Plasminogen Activator Receptor (suPAR) as a Predictor of Incident Atrial Fibrillation. J. Atr. Fibrillation.

[25] Borné Y., Persson M., Melander O., Smith J.G., Engström G. (2014). Increased plasma level of soluble urokinase plasminogen activator receptor is associated with incidence of heart failure but not atrial fibrillation. Eur. J. Heart Fail..

[26] Gaborit F.S., Kistorp C., Kümler T., Hassager C., Tønder N., Iversen K., Hansen P.M., Kamstrup P.R., Faber J., Køber L. (2020). Diagnostic utility of MR-proANP and NT-proBNP in elderly outpatients with a high risk of heart failure: The Copenhagen heart failure risk study. Biomarkers.

[27] Geelhoed B., Börschel C.S., Niiranen T., Palosaari T., Havulinna A.S., Fouodo C.J.K., Scheinhardt M.O., Blankenberg S., Jousilahti P., Kuulasmaa K. (2020). Assessment of causality of natriuretic peptides and atrial fibrillation and heart failure: A Mendelian randomization study in the FINRISK cohort. EP Eur..

[28] Öner Ö., Deveci F., Telo S., Kuluöztürk M., Balin M. (2020). MR-proADM and MR-proANP levels in patients with acute pulmonary embolism. J. Med. Biochem..

[29] De Marchis G.M., Schneider J., Weck A., Fluri F., Fladt J., Foerch C., Mueller B., Luft A., Christ-Crain M., Arnold M. (2018). Midregional proatrial natriuretic peptide improves risk stratification after ischemic stroke. Neurology.

[30] Schweizer J., Arnold M., König I.R., Bicvic A., Westphal L.P., Schütz V., Inauen C., Scherrer N., Luft A., Galovic M. (2022). Measurement of Midregional Pro-Atrial Natriuretic Peptide to Discover Atrial Fibrillation in Patients with Ischemic Stroke. J. Am. Coll. Cardiol..

[31] Lipinska-Gediga M., Mierzchala M., Durek G. (2012). Pro-atrial natriuretic peptide (pro-ANP) level in patients with severe sepsis and septic shock: Prognostic and diagnostic significance. Infection.

[32] ten Freyhaus H., Dumitrescu D., Schnorbach S., Kappert K., Viethen T., Hellmich M., Hunzelmann N., Rosenkranz S. (2015). CT-proET1 predicts pulmonary hemodynamics in Scleroderma-associated pulmonary hypertension. Clin. Res. Cardiol..

[33] Fietta A., Bardoni A., Salvini R., Passadore I., Morosini M., Cavagna L., Codullo V., Pozzi E., Meloni F., Montecucco C. (2006). Analysis of bronchoalveolar lavage fluid proteome from systemic sclerosis patients with or without functional, clinical and radiological signs of lung fibrosis. Arthritis Res. Ther..

[34] Andrukhova O., Salama M., Rosenhek R., Gmeiner M., Perkmann T., Steindl J., Aharinejad S. (2012). Serum glutathione S-transferase P1 1 in prediction of cardiac function. J. Card. Fail..

[35] Humbert M., Kovacs G., Hoeper M.M., Badagliacca R., Berger R.M.F., Brida M., Carlsen J., Coats A.J.S., Escribano-Subias P., Ferrari P. (2022). 2022 ESC/ERS Guidelines for the diagnosis and treatment of pulmonary hypertension: Developed by the task force for the diagnosis and treatment of pulmonary hypertension of the European Society of Cardiology (ESC) and the European Respiratory Society (ERS). Endorsed by the International Society for Heart and Lung Transplantation (ISHLT) and the European Reference Network on rare respiratory diseases (ERN-LUNG). Eur. Heart J..

[36] Medsger Jr T., Silman A., Steen V., Black C., Akesson A., Bacon P., Harris C., Jablonska S., Jayson M., Jimenez S. (1999). A disease severity scale for systemic sclerosis: Development and testing. J. Rheumatol..

[37] Valentini G., Iudici M., Walker U.A., Jaeger V.K., Baron M., Carreira P., Czirják L., Denton C.P., Distler O., Hachulla E. (2017). The European Scleroderma Trials and Research group (EUSTAR) task force for the development of revised activity criteria for systemic sclerosis: Derivation and validation of a preliminarily revised EUSTAR activity index. Ann. Rheum. Dis..

